# Transcriptome analysis reveals the host response to Schmallenberg virus in bovine cells and antagonistic effects of the NSs protein

**DOI:** 10.1186/s12864-015-1538-9

**Published:** 2015-04-19

**Authors:** Anne-Lie Blomström, Quan Gu, Gerald Barry, Gavin Wilkie, Jessica K Skelton, Margaret Baird, Melanie McFarlane, Esther Schnettler, Richard M Elliott, Massimo Palmarini, Alain Kohl

**Affiliations:** Section of Virology, Department of Biomedical Sciences and Veterinary Public Health, Swedish University of Agricultural Sciences, 750 07 Uppsala, Sweden; MRC-University of Glasgow Centre for Virus Research, Glasgow G61 1QH, Scotland, UK; UCD Veterinary Science Centre, School of Veterinary Medicine, University College Dublin, Belfield Dublin, Ireland

**Keywords:** Schmallenberg virus, Orthobunyavirus, RNA-seq, Transcriptome, Antiviral response, Non-structural protein NSs

## Abstract

**Background:**

Schmallenberg virus (SBV) is a member of the *Orthobunyavirus* genus (*Bunyaviridae* family) causing malformations and abortions in ruminants. Although, as for other members of this family/genus, the non-structural protein NSs has been shown to be an interferon antagonist, very little is known regarding the overall inhibitory effects and targets of orthobunyavirus NSs proteins on host gene expression during infection. Therefore, using RNA-seq this study describes changes to the transcriptome of primary bovine cells following infection with Schmallenberg virus (SBV) or with a mutant lacking the non-structural protein NSs (SBVdelNSs) providing a detailed comparison of the effect of NSs expression on the host cell.

**Results:**

The sequence reads from all samples (uninfected cells, SBV and SBVdelNSs) assembled well to the bovine host reference genome (on average 87.43% of the reads). During infection with SBVdelNSs, 649 genes were differentially expressed compared to uninfected cells (78.7% upregulated) and many of these were known antiviral and IFN-stimulated genes. On the other hand, only nine genes were differentially expressed in SBV infected cells compared to uninfected control cells, demonstrating the strong inhibitory effect of NSs on cellular gene expression. However, the majority of the genes that were expressed during SBV infection are involved in restriction of viral replication and spread indicating that SBV does not completely manage to shutdown the host antiviral response.

**Conclusions:**

In this study we show the effects of SBV NSs on the transcriptome of infected cells as well as the cellular response to wild type SBV. Although NSs is very efficient in shutting down genes of the host innate response, a number of possible antiviral factors were identified. Thus the data from this study can serve as a base for more detailed mechanistic studies of SBV and other orthobunyaviruses.

## Background

Schmallenberg virus (SBV) is a negative-sense, single-stranded, segmented, RNA virus belonging to the *Bunyaviridae* family, within the genus *Orthobunyavirus*. The virus was discovered 2011 in connection with outbreaks of diarrhoea, reduced milk production and fever in cattle in Germany and the Netherlands [1] and has since the initial discovery rapidly spread to many European countries [2]. Although infection is often connected with milder symptoms it can cause foetal abortions and malformations in ruminants [1,3]. SBV was the first orthobunyavirus detected in Europe, but the genus consists of over 170 viruses grouped into 18 serogroups and many of these are important pathogens. In humans, this includes for example La Crosse virus (LACV) causing encephalitis and Ngari Virus causing haermorrhagic fever [4]. Animal pathogens, apart from SBV, includes Akabane virus (AKBV) and Cache Valley virus (CVV) that also infect ruminants and can cause abortions and congenital malformations.

The SBV genome consists of three segments (L, large; M, medium; and S, small) coding for four structural proteins (N, nucleoprotein; Gn and Gc, glycoproteins; L, RNA-dependent RNA polymerase) and two non-structural proteins (NSm and NSs). Through the production of recombinant viruses lacking NSs (LACVdelNSs, AKBVdelNSs and Bunyamwera(BUNV)delNSs), NSs has been shown to be a major virulence factor for orthobunyaviruses as these deletant viruses are attenuated and show reduced growth rate compared to wild type virus [5-7]. SBV NSs has also been shown to be able to counteract host antiviral responses. Recombinant SBV lacking ability to express NSs (SBVdelNSs) is a potent inducer of type I IFN while SBV does not induce the IFN response after infection [8,9]. Apart from interfering with the IFN pathway, SBV NSs has also been shown to induce degradation of the RPB1 subunit of RNA polymerase II and to potentially have a pro-apoptotic role [10]. However, little is known about the overall inhibitory effects and targets of orthobunyavirus NSs proteins on gene expression in infected cells. Therefore, in this study we analysed the changes in the transcriptome of primary bovine cells following infection with either wild type SBV or SBVdelNSs (5, 6).

## Results and discussion

### Viral infection and sequencing output – quality and reference assembly

**Figure 1:**
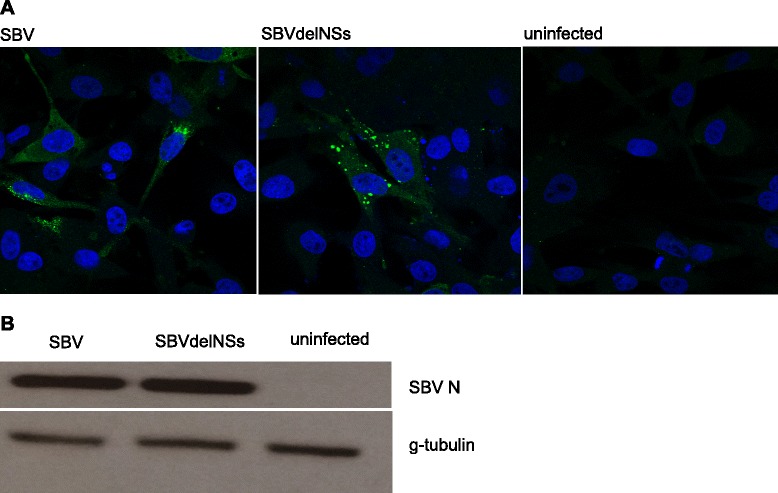
Infection of SBV and SBVdelNSs in bovine primary cells. **(A)** Immunofluorescent detection (N protein) of SBV and SBVdelNSs infected cells 16 h post infection (p.i.) showed about 30% infection rate. **(B)** Western blot analysis of SBV N protein and γ-tubulin at 16 h p.i.

We carried out RNA-seq analysis in infected and mock-infected primary bovine cells, in order to use cells with an intact cell-autonomous innate immune system. Bovine primary fibroblasts were infected with either SBV, SBVdelNSs or mock-infected and RNA was extracted at 16 h p.i.. Three biological replicates were assesses for each sample and cells were also infected in parallel for immunofluorescence (Figure 1A) and Western blot (Figure 1B) in order to confirm that the bovine primary cells were infected by SBV and SBVdelNSs at 16 h. Both the SBV and the SBVdelNSs infected cells were positive for the virus with an estimated infection rate at about 30%.

The samples for transcriptome analysis were sequenced on Illumina MiSeq. On average, 26.5 million reads/sample were generated with a phred quality value of 30 or higher and 87.43% of the sequence reads assembled against the *Bos taurus* genome (Ensembl Btau_4.0) using TopHat2 [11]. mRNA enrichment was carried out prior to sequencing and bunyaviruses in general (including SBV) lack a poly-A tail in their genome and mRNAs, consequently no assembly against the virus genome was performed.

### Differential expression analysis

**Figure 2:**
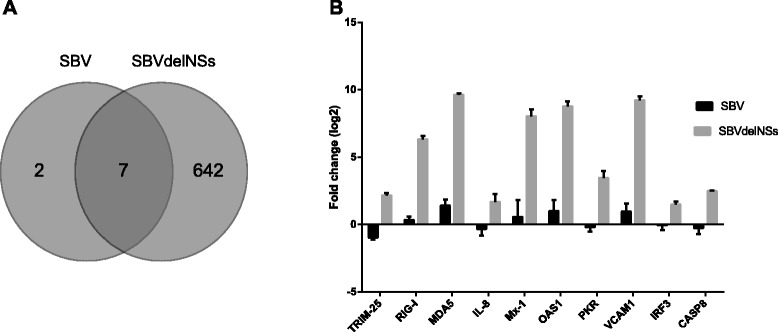
Analysis of differentially expressed (DE) genes 16 h p.i. **(A)** Venn diagram of the DE genes. **(B)** Quantitative PCR confirmation of transcript level changes detected in the RNA-seq DE analysis. For all genes there is a significant statistical difference (p ≤ 0.05) in gene expression between SBVdelNSs infection and SBV infected cells.

Cuffdiff2 [12] was used to identify differentially expressed (DE) genes (genes with a +/− 2-fold change or more and with p ≤ 0.05 were considered significant). 651 DE genes were identified and most were found in the SBVdelNSs infected cells (Figure 2A). Hence, the majority of the DE genes are as a direct or indirect result of the loss of NSs. Most (78.7%) of the DE genes affected by the loss of NSs were upregulated and fold differences ranged from 12.7-fold to the 1-fold cut-off value (on a log2 scale). The fold changes for down-regulated genes (21.3%) were more subtle, ranging from 2.72-fold to the 1-fold cut-off (log2 scale). The DE sequence analysis was validated for 10 genes using Sybr green realtime PCR (Figure 2B); the fold changes for SBVdelNSs compared to the SBV infected cells were all significant (p ≤ 0.05) and corresponded to the sequencing data.

### Pathway analysis

**Figure 3:**
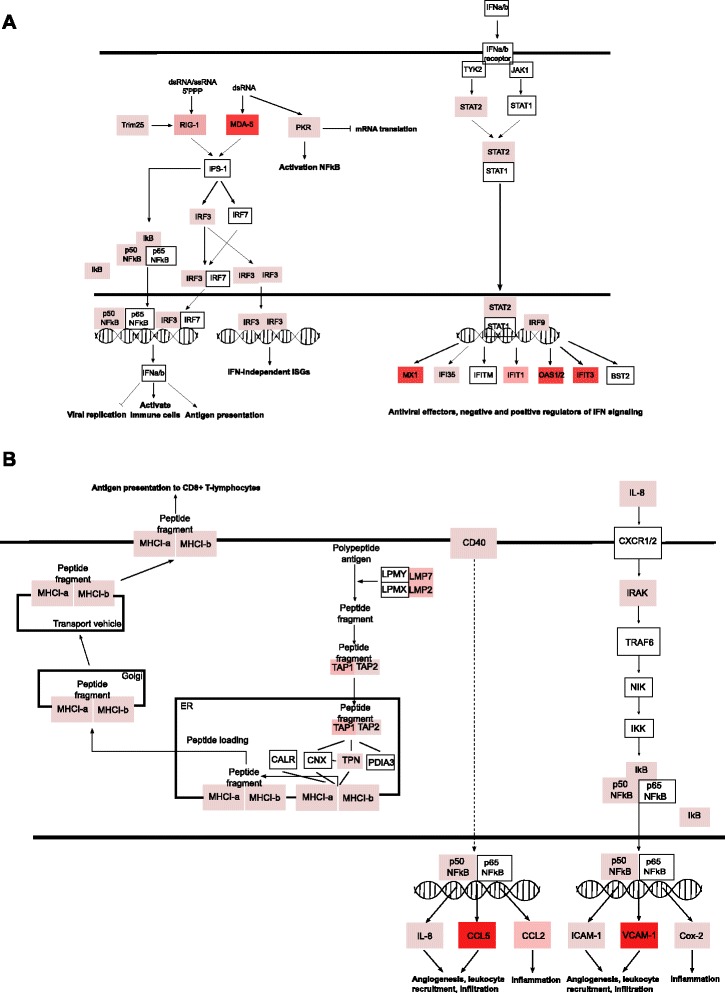
Schematic overview of some of the most significant IPA-identified, canonical host response pathways. **(A)** Role of pattern recognition pathways and the interferon signaling pathway. **(B)** Antigen presentation pathways and pathways involved in leukocyte recruitment and IL-8 signaling. Red boxes are genes upregulated in SBVdelNSs compared to uninfected cells; white boxes are genes not differentially expressed.

Ingenuity Pathway Analysis (IPA) (http://www.ingenuity.com/products/ipa) showed that the DE genes are, to a great extent, involved in pathways associated with host antiviral responses (Table 1), such as type I IFN-signalling and IFN-dependent gene expression, as well as pattern recognition. This is also evident when extracting the top 30 most upregulated genes (Table 2) as most of these have antiviral functions. The major molecules involved in viral RNA recognition including DDX58 (RIG-I), TRIM25, IFIH1 (MDA5), PKR, TLR3 were highly up-regulated in SBVdelNSs infected cells (Figure 3A), as are those involved in antigen presentation to CD8+ T-lymphocytes including MHC I α/β and TAP 1/2 (Figure 3B). As a consequence of activation of the viral RNA recognition pathways many interferon stimulated genes such OAS1/2, MX1 and several guanylate binding proteins, were upregulated [13-16]. Several interleukins were found among the DE transcripts, for example IL-8 and its downstream molecules (e.g. VCAM-1, ICAM-1 and Cox2) involved in inflammation, activation of angiogenesis and leucocyte infiltration/activation (Figure 3B).

**Table 1:** **Canonical pathway associated with infection of Schmallenberg virus or a mutant lacking NSs**

**SBV vs uninfected**			
**Ingenuity canonical pathways**	**-log(p-value)**	**Ratio**	**Genes**
Interferon Signaling	3.96E00	5.56E-02	OAS1,MX1
Activation of IRF by Cytosolic Pattern Recognition Receptors	3.42E00	2.74E-02	IFIT2,ISG15
Role of Pattern Recognition Receptors in Recognition of Bacteria and Viruses	3.06E00	1.83E-02	OAS1,OAS2
Role of Lipids/Lipid Rafts in the Pathogenesis of Influenza	1.95E00	3.45E-02	RSAD2
Ephrin B Signaling	1.47E00	1.22E-02	GNAL
CDK5 Signaling	1.39E00	1.03E-02	GNAL
G Beta Gamma Signaling	1.39E00	8.26E-03	GNAL
IL-1 Signaling	1.37E00	9.17E-03	GNAL
Androgen Signaling	1.29E00	6.9E-03	GNAL
Relaxin Signaling	1.2E00	6.1E-03	GNAL
**SBVdelNSs vs uninfected**			
**Ingenuity Canonical Pathways**	**-log(p-value)**	**Ratio**	**Genes**
Interferon Signaling	9.77E00	3.33E-01	IFIT3,SOCS1,IFIT1,OAS1,MX1,IFI35,STAT2,IRF9,PSMB8,JAK2,TAP1,IRF1
Activation of IRF by Cytosolic Pattern Recognition Receptors	9.32E00	2.05E-01	ZBP1,IRF9,TBK1,IL6,IRF3,ADAR,NFKB1,ISG15,IFIH1,NFKBIA,CD40,DDX58,STAT2,IFIT2,NFKBIB
Role of Pattern Recognition Receptors in Recognition of Bacteria and Viruses	8.35E00	1.56E-01	OAS1,PRKCQ,C3,OAS2,IL6,CCL5,IRF3,NFKB1,RNASEL,TLR2,IFIH1,TLR4,DDX58,EIF2AK2,TLR3,RIPK2,ATM
NF-κB Signaling	7.81E00	1.22E-01	AZI2,PRKCQ,RELB,IL36A,TNFAIP3,TBK1,IRAK3,NFKB1,TLR2,TLR4,TNIP1,NFKBIA,CD40,NGFR,MAP3K8,TRAF5,EIF2AK2,TLR3,NFKBIB,CASP8,ATM,TNFRSF11B
Role of Macrophages, Fibroblasts and Endothelial Cells in Rheumatoid Arthritis	7.74E00	8.77E-02	SOCS1,ICAM1,IL1RL1,IL6,CCL5,JAK2,NFKB1,FCGR1A,IL18R1,Prss2,NFKBIA,CCL2,NGFR,TLR3,TRAF5,NFKBIB,ATM,TNFRSF11B,IL8,VCAM1,PRKCQ,WNT2B,IL15,IL36A,IRAK3,IL7,TLR2,IL16,TLR4,CSF1
Dendritic Cell Maturation	7.58E00	1.04E-01	B2M,ICAM1,HLA-A,RELB,IL15,IL36A,HLA-DMB,IL6,JAK2,NFKB1,FCGR1A,TLR2,TLR4,NFKBIA,CD40,NGFR,STAT2,TLR3,NFKBIB,IL23A,ATM,TNFRSF11B
Antigen Presentation Pathway	6.97E00	2.38E-01	B2M,PSMB9,NLRC5,HLA-A,HLA-DMB,PSMB8,TAP1,TAP2,TAPBP,MR1
Role of PKR in Interferon Induction and Antiviral Response	6.62E00	2.04E-01	NFKBIA,TRAF5,EIF2AK2,TLR3,CASP8,NFKBIB,NFKB1,RNASEL,FCGR1A,IRF1
LXR/RXR Activation	6.08E00	1.15E-01	C3,IL1RL1,IL36A,IRF3,IL6,NFKB1,ABCA1,TLR4,CCL2,NGFR,MYLIP,LPL,CD14,PTGS2,TLR3,TNFRSF11B
Hepatic Fibrosis/Hepatic Stellate Cell Activation	5.93E00	1.1E-01	IL8,VCAM1,ICAM1,IL1RL1,IGFBP5,IL6,CCL5,NFKB1,TLR4,CD40,CCL2,CSF1,HGF,NGFR,TGFB2,CD14,TNFRSF11B

This table shows the top 10 IPA canonical pathways associated with SBV and SBVdelNSs infection, the p-value of Fisher’s exact test, the ratio (number of the genes in the dataset mapping to each specific pathway divided by the total number of genes in the pathway) and lists the DE genes from this study involved in respective pathway.

**Table 2:** **Up-regulated genes in SBV or SBVdelNSs infected cells**

**Top up-regulated genes**			
**Symbol**	**Entrez gene name**	**Fold change (log2)**	
		**SBV**	**delNSsSBV**
RSAD2	radical S-adenosyl methionine domain containing 2	1.96	11.518
ISG15	ISG15 ubiquitin-like modifier	1.86	10.886
IFIH1/MDA5	interferon induced with helicase C domain 1	-	9.987
GBP5	guanylate binding protein 5	-	9.837
OAS1	2′-5′-oligoadenylate synthetase 1, 40/46 kDa	2.24	9.818
IFIT2	interferon-induced protein with tetratricopeptide repeats 2	1.06	9.643
IFI27	interferon, alpha-inducible protein 27	-	9.387
ZBP1	Z-DNA binding protein 1	-	9.372
GBP4	guanylate binding protein 4	-	9.354
VCAM1	vascular cell adhesion molecule 1	-	9.287
OAS2	2′-5′-oligoadenylate synthetase 2, 69/71 kDa	2.41	9,.064
IFIT3	interferon-induced protein with tetratricopeptide repeats 3	-	8.87
MX1	myxovirus (influenza virus) resistance 1,	1.45	8.683
XAF1	XIAP associated factor 1	-	8.264
CCL5	chemokine (C-C motif) ligand 5	-	8.081
RTP4	receptor (chemosensory) transporter protein 4	-	8.059
GBP1	guanylate binding protein 1, interferon-inducible	-	7.483
GBP2	guanylate binding protein 2, interferon-inducible	-	6.853
LGALS9	lectin, galactoside-binding, soluble, 9	-	6.571
USP18	ubiquitin specific peptidase 18	-	6.536
CMPK2	cytidine monophosphate (UMP-CMP) kinase 2, mitochondrial	-	6.52
UBA7	ubiquitin-like modifier activating enzyme 7	-	6.15
DDX58	DEAD (Asp-Glu-Ala-Asp) box polypeptide 58	-	6.022
CXCL2	chemokine (C-X-C motif) ligand 2	-	5.946
CYP2J2	cytochrome P450, family 2, subfamily J, polypeptide 2	-	5.936
IFI6	interferon, alpha-inducible protein 6	-	5.807
MCHR1	melanin-concentrating hormone receptor 1	-	5.762
IFIT1	interferon-induced protein with tetratricopeptide repeats 1	-	5.681
HERC6	HECT and RLD domain containing E3 ubiquitin protein ligase family member 6	-	5.612
BATF2	basic leucine zipper transcription factor, ATF-like 2	-	5.483
GNAL	Guanine nucleotide-binding protein G(olf) subunit alpha	1.37	-
RPS3A	ribosomal protein S3A	1.16	-

The table indicates the most up-regulated genes during SBV and SBVdelNSs infection of bovine primary cells at 16 h (compared to uninfected cells). The fold-change is shown in log2 and “-” denotes no fold-change.

In SBV-infected cells only nine DE genes (RSAD2, ISG15, OAS1, OAS2, IFIT2, MX1, GNAL, RPS3A and MDFI) were identified (eight upregulated and one down-regulated) (Figure 2A and Table 1). The majority of these genes are involved in antiviral responses. All up-regulated genes in SBV infected cells, except guanine nucleotide binding protein (G protein), alpha activating activity polypeptide, olfactory type (GNAL) and ribosomal protein S3A (RPS3A), were also upregulated following SBVdelNSs infection, although to a much higher extent, indicating that NSs may not be able to completely shutdown all genes of the host antiviral response. Viperin (RSAD2) can be induced by a number of different viruses such as sindbis virus, Japanese encephalitis virus and lassa fever virus (LASV) either dependently and independently of IFN [17]. For example, Zapata JC *et al.* [18] showed that LASV strongly induce viperin early in infection (PBMC) while the attenuated ML29 has a weaker and delayed viperin induction suggesting that ML29 has a mechanism to interfere with the host-response signalling pathways. Antiviral activity of RSAD2 has been shown against for example BUNV, chikungunya virus, influenza virus and dengue virus through different mechanisms such as inhibition of viral replication and budding/egress [13-16,19]. MX1 has also been shown to have antiviral activity against a number of different RNA viruses such influenza and bunyaviruses [20]. MX1 is significantly upregulated in foetuses infected with CVV and it has been proposed to be involved in the clearance of the virus [21]. During LACV infection MX1 can bind to the nucleocapsid and inhibit viral replication [22]. Also, ISG15 is upregulated during foetal infection with CVV [21]. The OAS proteins performs their antiviral activity through the activation of RNAseL which leads to degradation of cellular and viral RNA [23] and OAS1, but not OAS2, is reported to contribute to a slight inhibition of BUNV and BUNVdelNSs viruses [19]. Hence although very few genes were up regulated during SBV infection most of the upregulated genes have all been previously shown to have antiviral activity for other similar viruses indicating that these genes, individually or collectively, may have an important role in the antiviral response and the consequent restriction of SBV infection. However, it should be noted that we only investigated host responses at one time point, and it is possible that at an earlier or later time point during the viral replication cycle, more DE genes may be found, as shown for dengue virus for example [24,25]. As not all primary cells were infected by SBV or SBVdelNSs it is possible that weak host responses to SBV might be masked by transcripts from uninfected cells. This may also explain why the DE analysis of SBV infected cells did not show a general host transcription shutdown as described [10], although mRNA turnover and cell types used in this and other studies may be further reasons. Only one transcript (MDFI – MyoD family inhibitor) was down-regulated in SBV infected cells compared to uninfected cells. This gene was also down-regulated in SBVdelNSs-infected cells, indicating that this change is not due to the NSs protein alone. This is a transcription factor that negatively regulates myogenic proteins. Knockout studies of the murine homolog, inhibitory of myogenic family (l-mfa), have shown that lack of l-mfa can lead to embryonic lethality and placental defects as well as skeletal patterning defects [26]. Although, the high viral levels of SBV seen in brain tissue are likely to cause the teratogenic effects seen during SBV infection the down-regulation of MDFI in SBV and SBVdelNSs infected cells is potentially of interest in relation to foetal abnormalities linked to SBV infection. Future studies will determine whether SBV and SBVdelINSs affect expression of MDFI in fetal brain tissue.

## Conclusions

We have investigated the effect of SBV infection on the host transcriptome and more specifically the effects induced by lack of NSs expression. Our results show the response to orthobunyavirus infection in a relevant host cell system and that NSs is very efficient in shutting down the immune response of the host but that despite this a number of known antiviral proteins (such as viperin, MX1, OAS1/2 etc.) are still induced during SBV infection. The data from this study serves to identify possible antiviral factors and serve as a basis for more detailed mechanistic studies of SBV and other orthobunyaviruses as well as the mechanism(s) of action of their NSs protein.

## Methods

### Cells

Bovine fibroblast cells were isolated from cow aortas by collagenase treatment using a method adapted described earlier [27]. Aortas were harvested from killed animals sourced from an abattoir and incubated at room temperature for 3 h in Dulbecco’s modified Eagles medium (DMEM) (Life Technologies) supplemented with 5 % foetal bovine serum (FBS), 100 U/ml penicillin 100 μg/ml streptomycin (P/S) and 2.5 μg/ml amphotericin B. After incubation, the aortas were cleaned, opened longitudinally and placed, intima layer down, into collagenase (2 mg/ml, DMEM) for 60 min at 37°C. After incubation the cells were isolated by collection of the collagenase supernatant and scraping of the aorta wall that was in contact with the collagenase. The supernatant containing the cells was centrifuged at 1000 rpm for 5 mins. The cells were then resuspended, seeded in 12-well plates and maintained at 37°C and 5% CO2 in DMEM with 20% FBS, 100 U/ml penicillin 100 μg/ml Streptomycin, 2.5 μg/ml amphotericin B. The population was initially a mixture of endothelial cells and fibroblasts but frequent passaging for approximately 10 days allowed the fibroblasts to outgrow the endothelial cells and form a pure population of fibroblasts.

### Viruses and infections

Rescued wild type SBV as well as a SBV lacking NSs described in Elliot *et al.* [8] were used throughout all the experiments.

Bovine fibroblasts were seeded in 24-well plates and at a confluence of 80% the cells were infected with SBV or SBVdelNSs at a MOI of 5 for 16 h. Mock-infected cells were included in parallel as control. For each condition three individual replicates were set up. Viral infection was confirmed by immunofluorescence and western blot targeting the N-protein of the virus. For western blot γ-tubulin (Sigma) was used as an internal control.

### RNA extraction

RNA was extracted from infected and uninfected cells using a combination of Trizol (Life Technology) and RNeasy Mini kit (Qiagen). In short, the cell media was discarded from the wells and the cells were mixed and homogenised in 750 μl Trizol. Chloroform (150 μl) was added and the sample mixed prior to a short incubation at room temperature and a centrifugation step at +4°C for 15 minutes. The upper aqueous phase was transferred to a new tube and mixed with 70% EtOH and then transferred to a RNeasy Mini Spin Column. The sample was bound to the column through centrifugation and then washed once with RW1 buffer and twice with RPE-buffer before eluted with 30 μl RNase free water. The RNA was stored at −80°C until further use.

### Library preparation and MiSeq sequencing

The TruSeq stranded mRNA sample preparation kit (Illumina) was used to enrich samples for mRNA and construct libraries for sequencing. Single end datasets with a read length of 150 nucleotides were generated from each sample library on an Illumina MiSeq. Each sample (SBV, SBVdelNSs and uninfected control) was set up in triplicate, and sequenced on a separate MiSeq run, with 9 runs being carried out in total. The sequences from the MiSeq runs have been deposited in the European Nucleotide Archive (ENA) and can be accessed through the study accession number PRJEB9007.

### Sequence quality and assembly

The FastQC software (http://www.bioinformatics.babraham.ac.uk/projects/fastqc) was used to check the RNA-Seq reads quality in order to remove low quality reads (Q > 30). The reads that passed the quality check were assembled against the *Bos taurus* genome (Ensembl Btau_4.0) using TopHat2 [11].

### Differential expression and pathway analysis

Cuffdiff2 within the Cufflink package (v2.2.1) [12] was used to identify differentially expressed (DE) genes and genes with a +/− 2-fold change or more and with p ≤ 0.05 were considered significant and used in the further analysis. Ingenuity Pathway Analysis (IPA) (http://www.ingenuity.com/products/ipa) was applied for the functional annotation and pathway analysis.

### Realtime PCR verification

150 ng RNA were converted into cDNA using superscript (Life Technologies) with 150 ng oligo(dT) (Promega) according to the manufacturer’s instructions. Fast SYBR Green Master mix (Life Technologies) was run according to the manufacturer’s protocol, primers are available on request. GAPDH was used as a housekeeping gene and the expression fold-changes was calculated using the 2^-∆∆Ct^ method.

## Abbreviations

SBV: Schmallenberg virus
LAVC: La Crosse virus
AKBV: Akabane virus
CCV: Cache Valley virus
BUNV: Bunyamwera
